# Correlation between single nucleotide polymorphisms of the ACTA2 gene and coronary artery stenosis in patients with type 2 diabetes mellitus

**DOI:** 10.3892/etm.2014.1510

**Published:** 2014-01-28

**Authors:** HUI FANG, XIAOLI LUO, YAN WANG, NIAN LIU, CHUNJIANG FU, HONGYONG WANG, YUQIANG FANG, WEIBIN SHI, YE ZHANG, CHUNYU ZENG, XUKAI WANG

**Affiliations:** 1 Department of Cardiology, Institute of War Surgery, The Third Affiliated Hospital of Third Military Medical University, Chongqing 400042, P.R. China; 2 Department of Medical Genetics, Third Military Medical University, Chongqing 400038, P.R. China

**Keywords:** type 2 diabetes mellitus, coronary artery stenosis, single nucleotide polymorphism

## Abstract

This study aimed to analyze the correlation between single nucleotide polymorphisms (SNPs) of the actin, aortic smooth muscle (ACTA2) gene and coronary artery stenosis in patients with type 2 diabetes mellitus (T2DM). Eight SNPs from the promoter region of the ACTA2 gene were screened. Patients with T2DM (n=251) were divided into two groups, those with severe coronary stenosis (SCS^+^ group; n=168) and those without severe coronary stenosis (SCS^−^ group; n=83). Patients were also divided according to lesion branching into those whose lesions involved less than three branches (LCA^−^ group) and those whose lesions involved at least three branches (LCA^+^ group). The clinical and laboratory data of the patients were collected, and the genotyping of eight SNPs was conducted followed by statistical analysis. Of the eight SNPs, only the rs1324551 SNP was identified to be significantly different between the SCS^+^ and SCS^−^ groups (P<0.05). The frequency of the rs1324551 G allele and GG genotype in the SCS^+^ group was significantly higher than that of the SCS^−^ group (P=0.044 and P=0.001, respectively). No significant difference was observed between the LCA^−^ and LCA^+^ groups. Following the deduction of age, gender and traditional risk factors, the odds ratios of the GG genotype in additive and recessive models were 2.93 [95% confidence interval (CI), 1.05–8.19; P=0.04] and 2.34 (95% CI, 1.09–5.02; P=0.03), respectively, and this relevancy was represented only in patients with low insulin levels. Age and smoking were also found to increase the risk of coronary artery lesions. In conclusion, the rs1324551 SNP in the promoter region of the ACTA2 gene was identified to be independently correlated with the degree of coronary artery stenosis in patients with T2DM and plasma insulin may inhibit coronary artery stenosis during the pathogenic process.

## Introduction

Transnational research (1) conducted by the World Health Organization has demonstrated that cardiovascular disease is the leading cause of mortality in patients with type 2 diabetes mellitus (T2DM). The percentage of fatalities caused by cardiovascular disease in the total number of mortalities is ~52%. Coronary heart disease (CHD) accounts for the largest proportion of cardiovascular diseases. Large-scale epidemiological surveys (2,3) have identified that the incidence and mortality of T2DM-associated CHD is increasing. Therefore, this type of disease has gradually become the focus of public health concerns. CHD commonly occurs in patients with T2DM. This is mainly due to a series of metabolic disorders, such as hypertension, hyperlipidemia, hyperglycemia and hyperinsulinemia based on insulin resistance. The role of insulin in inducing coronary artery lesions remains controversial. There are inconsistent findings at molecular and cellular levels in animal models and from the clinical results of different studies (4–9). Therefore, it is necessary to further investigate the role of insulin in coronary artery lesions in patients with T2DM.

The actin, aortic smooth muscle (ACTA2) gene encodes α-actin, a type of protein that is highly concentrated in matured vascular smooth muscle cells (VSMCs). It is composed of thin actin and thick myofilaments interaction, participating in vasodilation and vasoconstriction. Therefore, α-actin is a landmark protein of the contractile smooth muscle cell phenotype. It has been identified that the conversion of VSMCs from a contractile phenotype to a synthetic phenotype is accompanied by cell proliferation and migration, as well as marked reductions in the levels of α-actin and its mRNA (10). This conversion is important in the process of coronary artery lesion development (11,12), which also suggests that the ACTA2 gene is crucial for the etiology of coronary artery lesions.

CHD is considered to be a disease with multiple factors generated by the interaction of gene environments. Guo *et al* (13,14) conducted vascular pathology analysis and *in vitro* culture of VSMCs and found that the individuals with ACTA2 gene mutation are susceptible to a variety of vascular diseases caused by vasodilation or vascular blockage, including thoracic aortic aneurysm, the early-onset of CHD, stroke and moyamoya disease. As demonstrated in recent years, single nucleotide polymorphisms (SNPs) of the genes encoding adiponectin and tumor necrosis factor-α (TNF-α) may increase the risk of cardiovascular disease in patients with T2DM (15,16). It is speculated that certain SNPs in the promoter region of the ACTA2 gene may be involved in regulating the transcription of actin, thus changing the actin content in VSMCs for the phenotype conversion. These SNPs participate in the process of coronary atherosclerotic lesion development, accompanied by cell proliferation and migration. They may be associated with coronary artery lesions in patients with T2DM. In the present study, the correlation between SNPs in the promoter region of the ACTA2 gene and coronary artery stenosis in patients with T2DM was investigated, and the interaction of SNPs with plasma insulin was further analyzed.

## Subjects and methods

### Research subjects

Patients (n=251) with coronary angiography due to clinically diagnosed or suspected CHD in the Department of Cardiology, The Third Affiliated Hospital of Third Military Medical University (Chongqing, China) from March 2011 to August 2012 were semi-randomly selected for this study. T2DM was diagnosed according to the criteria issued by the American Diabetes Association in 2010 (17). Exclusion criteria were as follows: i) Patients with T1DM, secondary diabetes mellitus or serious organ disease; ii) patients with malignant tumors; iii) patients with a plasma C-peptide concentration of <0.6 ng/ml (18); and iv) patients who rejected participation in this study. Cases in which the stenosis degree of the main vessels (the left main coronary trunk, anterior descending branch, circumflex artery and right coronary artery) in angiography was >50% were defined to have severe coronary stenosis. The patients with T2DM (n=251) were divided into two groups, those with severe coronary stenosis (SCS^+^ group; n=168) and those without severe coronary stenosis (SCS^−^ group; n=83). Lesions involving any three of the left main coronary trunk (counted as double branches), anterior descending branch, circumflex artery and right coronary artery were defined as diffuse coronary artery lesions. The cases with lesions involving less than and at least three branches were included in the LCA^−^ and LCA^+^ groups, respectively.

Clinical and laboratory data of all patients were collected from the medical records. The clinical basic parameters included gender, age, smoking history, drinking history, blood pressure, blood lipids, glycosylated hemoglobin, fasting glucose, fasting insulin, C-peptide and coronary angiography results. Insulin resistance was assessed as follows: Homeostasis model assessment of insulin resistance (HOMA-IR) = fasting glucose (mmol/l) × fasting insulin (μU/ml)/22.5 (16). Patients were considered to have a smoking history if the number of cigarettes smoked in a lifetime was ≥100 (19), and a drinking history if the drinking frequency was at least one per month (20). The majority of patients had received antidiabetic, antihypertensive and lipid-lowering drugs, but the specific dosage and time were not obtained. Venous blood samples (2 ml) were obtained from each patient, and the genomic DNA was extracted and stored in a refrigerator at −80°C.

This study was approved by the ethics committee of The Third Affiliated Hospital of Third Military Medical University, and registration and verification was obtained from the Chinese Clinical Trial Registry Center (trial registration number: ChiCTR-OCS-12002158). Informed consents were obtained from the patients or the patients families.

### Screening of SNPs

Eight SNPs (rs6586164, rs10509561, rs1324551, rs4064, rs3740286, rs12775501, rs2028493 and rs3781211) were screened from the dbSNP (http://www.ncbi.nlm.nih.gov/snp), Ensembl genome (http://asia.ensembl.org/index.html), HapMap (http://hapmap.ncbi.nlm.nih.gov/) and UCSC (http://genome.ucsc.edu/) databases using bioinformatics technology. They were located in the promoter region of the ACTA2 gene in the Chinese Han population and the minor allele frequency (MAF) was ≥0.2 (Table I).

### Polymerase chain reaction (PCR) and genotyping

Genomic DNA in whole blood was extracted using a traditional salting-out method. According to the gene sequence (NG_011541.1), Primer 5.0 software (Premier Biosoft, Palo Alto, CA, USA) was used to design the primers of four sections containing eight SNPs and the sequencing primers are shown in Table II. The PCR system (50 μl) consisted of 1 μl genomic DNA, 2 μl primer, 22 μl deionized water and 25 μl 2X Taq Plus Master Mix (Sino Biotech Co., Ltd., Shanghai, China). The PCR conditions were as follows: i) Sections 1 and 2: Predenaturation at 95°C for 5 min, 35 cycles (degeneration at 95°C for 30 sec, renaturation at 55°C for 30 sec and elongation at 72°C for 1 min), elongation at 72°C for 5 min; ii) section 3: Predegeneration at 95°C for 5 min, 35 cycles (degeneration at 95°C for 30 sec, renaturation at 53°C for 30 sec and elongation at 72°C for 1 min), elongation at 72°C for 5 min; iii) section 4: Predegeneration at 95°C for 5 min, 35 cycles (degeneration at 95°C for 20 sec, renaturation at 50°C for 20 sec and elongation at 72°C for 30 sec), elongation at 72°C for 5 min. The PCR products were sequenced in the Beijing Huada Gene Research Center (Beijing, China) using a 3730 DNA analyzer (Applied Biosystems, Inc., Woburn, MA, USA).

### Statistical analysis

Statistical analysis was performed using SPSS software, version 13.0 (SPSS Inc., Chicago, IL, USA). As the vast majority of continuous data in this study were with a non-normal distribution, following logarithmic transformation, the Mann-Whitney U Test (nonparametric rank test) was used to analyze differences between the SCS^−^ and SCS^+^ groups. Chi-square test was performed to analyze the Hardy-Weinberg equilibrium and frequency difference of enumeration data between the two groups. Odds ratio (OR) values and 95% confidence intervals (CIs) of the degree of coronary artery stenosis with and without adjustment for age, gender and additional confounding factors, were analyzed by unrestricted logistical regression. All continuous and counting variables were represented as the median (25–75%) and percentage (%), respectively. P<0.05 was considered to indicate a statistically significant difference.

## Results

### Statistical indicators

General statistical indicators and important clinical biochemical indexes of the subjects are shown in Table III. In the SCS^+^ group, there were 110 (65.5%) cases of diffuse coronary artery lesions (LCA^+^). There were significant differences in age, gender and smoking history between the SCS^+^ and SCS^−^ groups (P≤0.01), but no significant differences in body mass index (BMI), blood pressure, blood lipid levels, glycosylated hemoglobin (GHbA1c), fasting insulin, HOMA-IR and other parameters were observed.

### Gentoype

Genotyping results (Table IV) showed that in the SCS^−^ group the frequencies of A and G alleles were 47.6% and 52.4%, respectively. This was in accordance with the Hardy-Weinberg equilibrium and the Chi-square test (P=0.253). Of the eight SNPs, only rs1324551 was associated with the degree of coronary stenosis in patients with T2DM (the statistical results of other SNPs are not shown). The frequencies of G alleles and the GG genotype in rs1324551 in the SCS^+^ group were 208 (61.9%) and 67 (39.9%), respectively, while in the SCS^−^ group they were 87 (52.4%) and 21 (25.3%), respectively. The frequency of G alleles and the GG genotype in the SCS^+^ group were significantly higher than those of the SCS^−^ group (P=0.044 and P=0.001, respectively). This indicates that the rs1324551 SNP may be closely associated with the degree of coronary stenosis. However, no significant differences in the frequency of G alleles and the GG genotype in rs1324551 were observed between the LCA^−^ and LCA^+^ groups, suggesting that the rs1324551 SNP may be closely associated with the degree of coronary artery stenosis, but not with the degree of diffuse lesions.

It was further conformed by unrestricted logistical regression analysis that following the deduction of confounding variables, such as age, gender, smoking history, blood pressure, BMI, GHbA1c and blood lipid levels, the rs1324551 SNP was still associated with an increased risk of CHD, with increased ORs and 95% CIs in patients with T2DM.

OR values and 95% CIs for rs1324551 in different gene models after the deduction of combined risk factors are shown in Table V. In the additive and recessive models, following the deduction of age and gender, the risk of severe coronary artery stenosis in patients carrying the GG genotype was 2.20-fold higher than in those carrying the AA genotype (95% CI, 0.98–4.93; P=0.057), while no significant difference was identified between patients carrying the AG genotype and those carrying the AA genotype. In addition, the risk of severe coronary artery stenosis in patients carrying the GG genotype was 2.01-fold higher than in those carrying the AA + AG genotype (95% CI, 1.10–3.66; P=0.02). Following the deduction of age, gender and traditional risk factors, the OR values for the GG genotype in the additive and recessive models were 2.89 (95% CI, 1.03–8.05; P=0.04) and 2.30 (95% CI, 1.07–4.92, P=0.03), respectively. Based on the deduction of age, gender and traditional risk factors, the effect of fasting insulin was deducted; the OR values for the GG genotype were 2.93 (95% CI, 1.05–8.19; P=0.04) and 2.34 (95% CI, 1.09–5.02; P=0.03) in the additive and recessive models, respectively. The dominant model indicated no significant difference in the risk of severe coronary stenosis between patients carrying the G allele and those not carrying it.

Similar to previous studies, the present study indicated that gender and smoking history independently increase the risk of severe coronary artery stenosis in patients with T2DM. In the recessive model, the adjusted OR values were 1.06 (95% CI, 1.02–1.20; P=0.007) and 10.59 (95% CI, 1.27–88.11; P=0.03), respectively (data not shown), but the effects of traditional risk factors in promoting coronary artery disease were not found.

These results suggest that the rs1324551 SNP was significantly associated with coronary artery stenosis in patients with T2DM and the risk of severe coronary artery stenosis in patients carrying the GG genotype was 1–2-fold higher than that of patients who were not carrying the GG genotype. In order to further investigate the interaction of the rs1324551 SNP with plasma insulin during the pathogenic process, the 251 patients were divided into a high insulin group and a low insulin group for stratified analysis based on the median fasting insulin concentration (9.54 μU/ml). The results of statistical analysis indicated that the coronary lesion-promoting effects of the rs1324551 SNP were represented only in the low insulin group. As shown in Table VI, the OR values in the additive, recessive and dominant models were 12.9 (95% CI, 1.72–97.15; P=0.01), 3.29 (95% CI, 0.98–11.00; P=0.054) and 6.81 (95% CI, 1.13–41.19; P=0.04), respectively. In the high insulin group, no significant risk of severe coronary artery disease was identified. This suggests that in the process of coronary artery disease in patients with T2DM, plasma insulin may interact with the rs1324551 SNP. An additional stratified analysis with gender, age and coronary stenosis degree was conducted and no significant pathogenic risks were found (P>0.05; data not shown). We speculate that the small sample size used for the stratified analysis caused a reduction of test efficiency.

## Discussion

The present study showed that the carrying rate of rs1324551 GG genotype in the SCS^+^ group was significantly higher than that of the SCS^−^ group, suggesting the probable correlation between the GG gene and coronary artery stenosis in patients with T2DM. Logistic regression analysis indicated that in additive and recessive models, following the deduction of age, gender and traditional risk factors, the GG genotype may have increased the risk of coronary stenosis in patients with T2DM. The AG genotype and the AG + GG genotype (dominant model) showed no evident effect on the risk of coronary stenosis. In the present study, the risk of coronary stenosis was only associated with the GG genotype, suggesting that the rs1324551 SNP may cause the disease with a recessive inheritance mode, but the pathogenesis of this SNP remain unclear. As found in the UCSC database, rs1324551 is located between two transcription factor binding sites in the promoter region at the 5′-UTR end of the ACTA2 gene. We speculate that in patients with T2DM, the GG genotype may decrease the affinity of the ACTA2 gene transcription factor to the binding sites on DNA, thus relatively inhibiting the volume of α-actin transcripts. A further mechanism may be a linkage disequilibrium between rs1324551 and certain functional SNPs at the 3′-UTR end of the ACTA2 gene, which accelerates the degradation of α-actin mRNA. Therefore, the expression levels of α-actin in VSMCs are decreased, which causes the phenotype conversion of VSMCs accompanied with cell proliferation and migration, ultimately promoting coronary artery stenosis. Guo *et al* (13) demonstrated that there were five mechanisms involved in the development of atherosclerotic lesions. The ACTA2 gene mutation may cause the proliferation of VSMCs resulting in coronary luminal stenosis. It has been found that there are a greater number of proliferated VSMCs and fewer lipocytes in the coronary artery lesion tissues of patients carrying a mutated ACTA2 gene (13). Unfortunately, due to ethical reasons, coronary artery lesion tissue was not obtained in the present. Therefore, the content of α-actin in VSMCs and the levels of its mRNA have yet to be detected. Our hypothesis requires further validation in cell and/or animal experiments.

Notably, in the logistic regression analysis, following the deduction of fasting insulin, the pathogenic risks of coronary artery stenosis in the additive and recessive models in patients with T2DM carrying the GG genotype were slightly increased. The stratified analysis according to fasting insulin level showed that the coronary artery stenosis-promoting effects of the rs1324551 SNP were only represented in the low insulin group, with no evident pathogenic risks in the high insulin group. This indicates that plasma insulin may have a protective role in coronary artery stenosis in patients with T2DM. At present, the role of insulin in coronary artery stenosis remains controversial. A previous study (4) proposed that insulin has anti-atherogenic effects, which are mainly achieved by lowering plasma fatty acid levels, anti-inflammatory and anti-embolism effects and the dilation of blood vessels. The *in vivo* anti-atherosclerotic effects of insulin are far greater than the pro-atherosclerosis effects. The present study was a single-center, case-controlled study, which selected patients with T2DM as subjects. The results were in accordance with the study by Breen and Giacca (4). However, additional large-scale studies with improved experimental designs are required to further verify the role of insulin in coronary artery disease. The stratified analysis according to gender, age and degree of coronary artery stenosis identified no significant effects of the rs1324551 SNP in the promotion of coronary artery stenosis. This may be due to the small sample size used in the stratified analysis.

The present study also demonstrated that in the SCS^+^ group, there were 110 (65.5%) cases with lesions involving at least three vessel branches. This is in accordance with the diffuse characteristics of coronary artery disease in patients with T2DM (21). There were no significant differences in the GG genotype carrying rate between the LCA^−^ and LCA^+^ groups, and the logistical regression analysis showed no correlation between the rs1324551 SNP and diffuse coronary artery lesions. This indicates that the rs1324551 SNP does not participate in the process of diffuse coronary artery disease in patients with T2DM. In addition, these results support the speculation that the rs1324551 GG genotype may cause coronary artery stenosis by promoting the proliferation of VSMCs. In accordance with the results of previous studies (22,23), old age and smoking history were found to increase the risk of coronary artery disease in patients with T2DM in the present study, but the effects of other traditional risk factors that promote coronary artery disease were not observed. This may be due to the fact that the majority of the subjects in the present study were patients receiving therapeutic agents (Table I), and the blood pressure, blood glucose, blood lipid levels and other risk factors have been artificially adjusted to normal values; therefore, their effects were difficult to observe. However, age and smoking status are not greatly variable in a short term study, so their correlation with the risk of coronary stenosis was able to be observed.

In conclusion, to the best of our knowledge, this study is the first to demonstrate a significant correlation between the rs1324551 GG genotype in the ACTA2 gene and coronary artery stenosis in patients with T2DM. The possible molecular mechanism may be that the GG genotype is positioned in the non-coding region of the ACTA2 gene and may regulate the transcription level of the ACTA2 gene, thus inhibiting the expression of α-actin. This would cause phenotype conversion in VSMCs accompanied by cell proliferation and migration, thereby promoting coronary artery stenosis in patients with T2DM. In addition, this study showed that the coronary arteries in patients with T2DM were characterized by diffuse lesions and that insulin induced an inhibiting effect on coronary artery stenosis. However, the small sample size, and the lack of multi-center research data and experiments investigating the mechanisms of SNP function are the limitations of this study.

## Figures and Tables

**Table I:** Information associated with SNPs.

SNPs	MAF	Source from CHB	Chromosome position
rs6586164	0.38 (A/T)	Yes	90752031
rs10509561	0.24 (A/T)	No	90751912
rs1324551	0.38 (T/C)	Yes	90751516
rs4064	0.24 (C/G)	No	90751380
rs3740286	0.37 (C/G)	Yes	90751340
rs12775501	0.43 (C/T)	Yes	90750909
rs2028493	0.22 (A/C)	Yes	90696214
rs3781211	0.32 (G/T)	Yes	90695265

SNPs, single nucleotide polymorphisms; MAF, minimum allele frequency; CHB, Chinese Han population in Beijing.

**Table II:** Primer and product information of eight SNPs in the ACTA2 gene.

Gene sections	SNP loci	Primer	Length (bp)
Section 1	rs6586164, rs10509561, rs1324551	Sense: 5′-GGTAAAGGTGCTATTGGT-3′	
		Antisense: 5′-AACTGCCTTGTCTCCCTT-3′	903
Section 2	rs4064, rs3740286, rs12775501	Sense: 5′-AAGAAAGGGCAAAAGAAG-3′	
		Antisense: 5′-CTCAGAACGCTGGAGGAC-3′	598
Section 3	rs2028493	Sense: 5′-GGCTGTTCTTGCGTTGCT-3′	
		Antisense: 5′-TGGGTTCTCCCAGGTGGT-3′	520
Section 4	rs3781211	Sense: 5′-TGGGTGAAAAGTGGTAGT-3′	
		Antisense: 5′-CAAGATGAAAAAGAATGG-3′	462

SNPs, single nucleotide polymorphisms; ACTA2, actin, aortic smooth muscle.

**Table III:** Clinical parameters of the study subjects (n=251).

Parameter	SCS^−^ (n=83)	SCS^+^ (n=168)	P-value
Age (years)	63 (58–71)	68 (61–75)	0.01
Male gender, n (%)	35 (42.2)	101 (60.1)	0.01
Non-smoker, n (%)	79 (95)	136 (81.0)	<0.01
Non-drinker, n (%)	77 (92.8)	159 (94.6)	NS
No family history, n (%)	69 (83.1)	137 (81.5)	NS
BMI (kg/m^2^)	24.97 (22.89–26.44)	24.35 (22.67–26.37)	NS
SBP (mmHg)	130.00 (120.00–140.00)	130.00 (120.00–140.00)	NS
DBP (mmHg)	78.00 (70.00–82.00)	75.00 (70.00–80.00)	NS
FG (mmol/l)	6.52 (5.54–8.13)	6.50 (5.26–7.90)	NS
FINS (uU/ml)	9.50 (5.79–13.39)	9.33 (5.76–15.58)	NS
HOMA-IR	2.47 (1.67–4.06)	2.51 (1.55–5.00)	NS
GHbA1c (%)	6.80 (6.50–7.50)	7 (6.40–8.30)	NS
C-peptide (ng/ml)	2.55 (1.93–3.22)	2.69 (2.04–3.56)	NS
TC (mmol/l)	4.29 (3.40–5.05)	4.23 (3.34–4.98)	NS
TG (mmol/l)	1.53 (1.12–2.50)	1.45 (1.01–2.10)	NS
HDL-C (mmol/l)	1.08 (0.89–1.29)	0.99 (0.85–1.18)	NS
LDL-C (mmol/l)	2.09 (1.68–2.58)	2.24 (1.72–2.65)	NS
LCA^+^, n (%)	3 (3.6)	110 (65.5)	NS

Values are presented as the median with the range in parentheses, unless otherwise indicated. BMI, body mass index; SBP, systolic blood pressure; DBP, diastolic blood pressure; FG, fasting glucose; FINS, fasting insulin; HOMA-IR, homeostasis model assessment of insulin resistance; GHbA1c, glycosylated hemoglobin; TC, total cholesterol; TG, total triglycerides; HDL-C, high-density lipoprotein cholesterol; LDL-C, low-density lipoprotein cholesterol; LCA^+^, lesion involving ≥3 branches; SCS^−^, without severe coronary stenosis; SCS^+^, with severe coronary stenosis; NS, no significant difference.

**Table IV:** rs1324551 allele and genotype frequencies in the study subjects (n=251).

Model	SCS^−^ (n=83)	SCS^+^ (n=168)	P-value	LCA^−^ (n=138)	LCA^+^ (n=113)	P-value
Allele model, n (%)						
A	79 (47.6)	128 (38.1)		119 (43.1)	88 (38.9)	
G	87 (52.4)	208 (61.9)	0.044	157 (56.9)	138 (61.1)	NS
Genotype model, n (%)						
AA	17 (20.5)	27 (16.1)		26 (18.8)	18 (15.9)	
GG	21 (25.3)	67 (39.9)	0.001	45 (32.6)	43 (38.1)	NS
AG	45 (54.2)	74 (44.0)		67 (48.6)	52 (46.0)	
Dominant model, n (%)						
GG + AG	66 (79.5)	141 (83.9)	0.384	112 (81.2)	95 (84.1)	NS
Recessive model, n (%)						
AA + AG	62 (74.7)	101 (60.1)	0.025	93 (67.4)	70 (61.9)	NS

SCS^−^, without severe coronary stenosis; SCS^+^, with severe coronary stenosis; LCA^−^, lesion involving <3 branches; LCA^+^, lesion involving ≥3 branches; NS, no significant difference.

**Table V:** ORs and 95% CIs for rs1324551 after the deduction of combined risk factors.

Deducted factors	Additive model AA/GG/AG	Recessive model GG/(AA + AG)	Dominant model AA/(AG + GG)
A + G	2.20 (0.98–4.93)^a^	2.01 (1.10–3.66)^b^	1.48 (0.73–2.98)
A + G + C	2.89 (1.03–8.05)^b^	2.30 (1.07–4.92)^b^	1.76 (0.74–4.23)
A + G + C + F	2.93 (1.05–8.19)^b^	2.34 (1.09–5.02)^b^	1.77 (0.74–4.26)

a P=0.057;

b P<0.05;

A, age; G, gender; C, traditional risk factors (smoking history, blood pressure, BMI, GHbA1c, blood lipid levels and HOMA-IR); F, fasting insulin. OR, odds ratio; CI, confidence interval; BMI, body mass index; GHbA1c, glycosylated hemoglobin; HOMA-IR, homeostasis model assessment of insulin resistance.

**Table VI:** Stratification analysis for rs1324551 according to fasting insulin level.

Group	Additive model AA/GG/AG	Recessive model GG/AA + AG	Dominant model AA/AG + GG
High insulin	0.96 (0.22–4.26)	1.34 (0.43–4.23)	0.73 (0.20–2.60)
Low insulin	12.9 (1.72–97.15)^b^	3.29 (0.98–11.00)^a^	6.81 (1.13–41.19)^b^

a P=0.054;

b P<0.05 compared with the high insulin group.

Deducted factors: Age, gender, smoking history, blood pressure, body mass index, glycosylated hemoglobin, blood lipid levels and homeostasis model assessment of insulin resistance. OR, odds ratio; CI, confidence interval; FINS, fasting insulin.
